# Toward an Atomic-Level Understanding of Ceria-Based Catalysts: When Experiment and Theory Go Hand in Hand

**DOI:** 10.1021/acs.accounts.1c00226

**Published:** 2021-06-17

**Authors:** Marc Ziemba, Christian Schilling, M. Verónica Ganduglia-Pirovano, Christian Hess

**Affiliations:** † Eduard-Zintl-Institute of Inorganic and Physical Chemistry, Technical University of Darmstadt, Alarich-Weiss-Str. 8, 64287 Darmstadt, Germany; ‡ Instituto de Catálisis y Petroleoquímica - Consejo Superior de Investigaciones Científicas, Marie Curie 2, 28049 Madrid, Spain

## Abstract

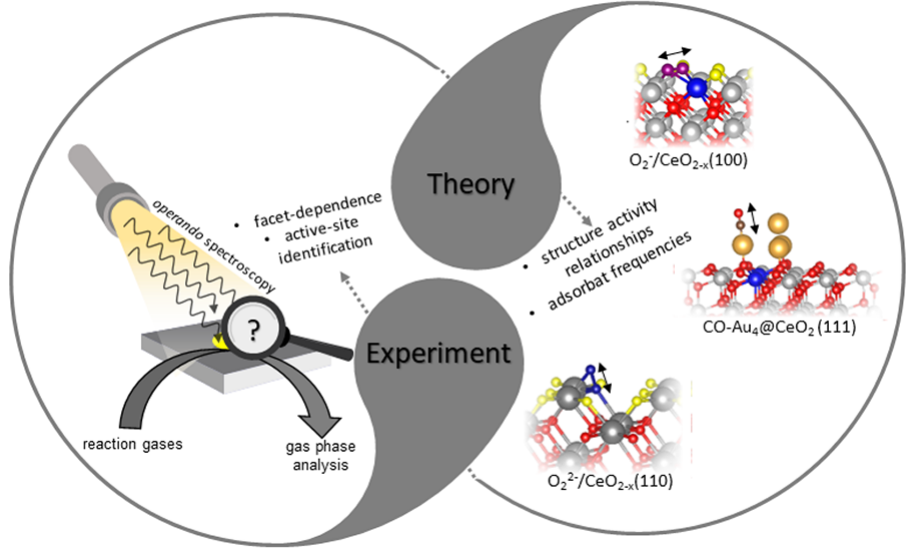

Because ceria (CeO_2_) is a key ingredient in the formulation of many catalysts, its catalytic roles have received a great amount of attention from experiment and theory. Its primary function is to enhance the oxidation activity of catalysts, which is largely governed by the low activation barrier for creating lattice O vacancies. Such an important characteristic of ceria has been exploited in CO oxidation, methane partial oxidation, volatile organic compound oxidation, and the water–gas shift (WGS) reaction and in the context of automotive applications. A great challenge of such heterogeneously catalyzed processes remains the unambiguous identification of active sites.

In oxidation reactions, closing the catalytic cycle requires ceria reoxidation by gas-phase oxygen, which includes oxygen adsorption and activation. While the general mechanistic framework of such processes is accepted, only very recently has an atomic-level understanding of oxygen activation on ceria powders been achieved by combined experimental and theoretical studies using *in situ* multiwavelength Raman spectroscopy and DFT.

Recent studies have revealed that the adsorption and activation of gas-phase oxygen on ceria is strongly facet-dependent and involves different superoxide/peroxide species, which can now be unambiguously assigned to ceria surface sites using the combined Raman and DFT approach. Our results demonstrate that, as a result of oxygen dissociation, vacant ceria lattice sites are healed, highlighting the close relationship of surface processes with lattice oxygen dynamics, which is also of technical relevance in the context of oxygen storage-release applications.

A recent DFT interpretation of Raman spectra of polycrystalline ceria enables us to take account of all (sub)surface and bulk vibrational features observed in the experimental spectra and has revealed new findings of great relevance for a mechanistic understanding of ceria-based catalysts. These include the identification of surface oxygen (Ce–O) modes and the quantification of subsurface oxygen defects. Combining these theoretical insights with *operando* Raman experiments now allows the (sub)surface oxygen dynamics of ceria and noble metal/ceria catalysts to be monitored under the reaction conditions.

Applying these findings to Au/ceria catalysts provides univocal evidence for ceria support participation in heterogeneous catalysis. For room-temperature CO oxidation, *operando* Raman monitoring the (sub)surface defect dynamics clearly demonstrates the dependence of catalytic activity on the ceria reduction state. Extending the combined experimental/DFT approach to *operando* IR spectroscopy allows the elucidation of the nature of the active gold as (pseudo)single Au^+^ sites and enables us to develop a detailed mechanistic picture of the catalytic cycle. Temperature-dependent studies highlight the importance of facet-dependent defect formation energies and adsorbate stabilities (e.g., carbonates). While the latter aspects are also evidenced to play a role in the WGS reaction, the facet-dependent catalytic performance shows a correlation with the extent of gold agglomeration. Our findings are fully consistent with a redox mechanism, thus adding a new perspective to the ongoing discussion of the WGS reaction.

As outlined above for ceria-based catalysts, closely combining state-of-the-art *in situ*/*operando* spectroscopy and theory constitutes a powerful approach to rational catalyst design by providing essential mechanistic information based on an atomic-level understanding of reactions.

## Key References

- SchillingC.; Ganduglia-PirovanoM. V.; HessC.Experimental and Theoretical Study on the Nature of Adsorbed Oxygen Species on Shaped Ceria Nanoparticles. J. Phys. Chem. Lett.2018, 9( (22), ), 6593–659810.1021/acs.jpclett.8b0272830373369.^1^*The shape-dependent adsorption and activation of oxygen on ceria nanoparticles with (111) and (100) facets is elucidated by in situ Raman spectroscopy and related to unique adsorption sites using DFT calculations.*
- ZiembaM.; Ganduglia-PirovanoM. V.; HessC.Elucidating the Oxygen Storage-Release Dynamics in Ceria Nanorods by Combined Multi-Wavelength Raman Spectroscopy and DFT. J. Phys. Chem. Lett.2020, 11, 8554–855910.1021/acs.jpclett.0c0260732970436.^2^*Ceria nanorods are shown to exhibit facet-dependent properties regarding oxygen activation, decomposition, and lattice oxygen dynamics, which are of great interest for oxygen storage–release functions.*
- ZiembaM.; HessC.Influence of Gold on the Reactivity Behaviour of Ceria Nanorods in CO Oxidation: Combining Operando Spectroscopies and DFT Calculations. Catal. Sci. Technol.2020, 10( (11), ), 3720–373010.1039/D0CY00392A.^3^*Structure–activity relations are established for CO oxidation over ceria and Au/ceria nanocubes/nanorods, elucidating the roles of (110) and (100) facet termination, employing operando spectroscopy combined with DFT calculations.*
- ZiembaM.; Ganduglia-PirovanoV.; HessC.Insight into the Mechanism of the Water-Gas Shift Reaction over Au/CeO_2_ Catalysts Using Combined Operando Spectroscopies. Faraday Discuss.2021, 229, 232–25010.1039/C9FD00133F33634801.^4^*The role of ceria termination in the mechanism of the WGS reaction over ceria-supported gold catalysts is elucidated by operando spectroscopy combined with isotope labeling and DFT calculations.*

## 1 Introduction

Heterogeneous catalysis is a key enabling technology for achieving an efficient and more sustainable utilization of resources. The rational design of better catalysts requires an atomic-level understanding of their mode of operation, including the identification and characterization of active sites. For model catalysts, methods for characterizing the structure of the catalytic surface have been developed (e.g., STM, AFM) and applied to oxide-based systems.^5,6^ However, the establishment of structure–activity relationships in catalysis using model catalysts remains challenging, and these methods cannot be applied in a straightforward manner to powder systems, the technologically relevant form of catalysts. Thus, new approaches are urgently needed to achieve a better and ultimately an atomic-level understanding of real-world catalysts.

Unravelling the working principle of catalysts requires the development and application of methods that enable the identification and characterization of active sites. To be of relevance, the structural analysis should be performed under real working conditions and in real time and should be combined with the simultaneous detection of activity (*operando* approach). However, the synergy between theory and experiment using model and powder catalysts is crucial to unravelling the structure of the working catalysts while bridging the materials and complexity gaps in catalysis. While efforts have already been made to enable an atomic-level characterization of active sites under *operando* conditions,^7^ further development is necessary.

Ceria (CeO_2_) is among the catalytically most active metal oxides, with particular redox properties and relatively high abundance. Ceria and ceria-containing materials are of great interest for environmental and energy conversion applications and thus have received a great deal of attention from experiment and theory. For instance, the low-temperature water–gas shift reaction, which increases the H_2_/CO ratio after steam reforming, is commonly run over Cu/ZnO/Al_2_O_3_ catalysts, which are pyrophoric and thermally unstable. Alternatively, reducible oxides such as ceria supporting low loadings of noble metals (Pt, Au) have been discussed.^8−10^ Moreover, ceria-based catalysts are used in automotive emission control^11^ and oxidation catalysis^3,4,8,12,13^ and are discussed in the context of fuel cell applications^14−16^ and biology.^17−19^ Furthermore, ceria is a reducible oxide, the intrinsic physical and chemical properties of which are current research topics on their own.

The booming interest in ceria, particularly in catalysis, is reflected in the number of review articles in the literature^5,15,16,20−31^ addressing the surface chemistry of ceria, its defect structure, and the relation of both to catalytic properties. While in those earlier works either an experimental^5,15,16,21,22,25−29^ or a theoretical^20,23,24,30,31^ perspective has been taken, there has been no account of the close combination and interplay of *in situ*/*operando* spectroscopy and theory and its potential to gain an atomic-level understanding of ceria and ceria-based powder catalysts. To this end, we hope that our account triggers new research activities and discussions in the context of ceria catalysis but also regarding other catalytically relevant and/or reducible oxide materials such as TiO_2_ and V_2_O_5_.

## 2 Structure of Ceria

**Figure 1:**
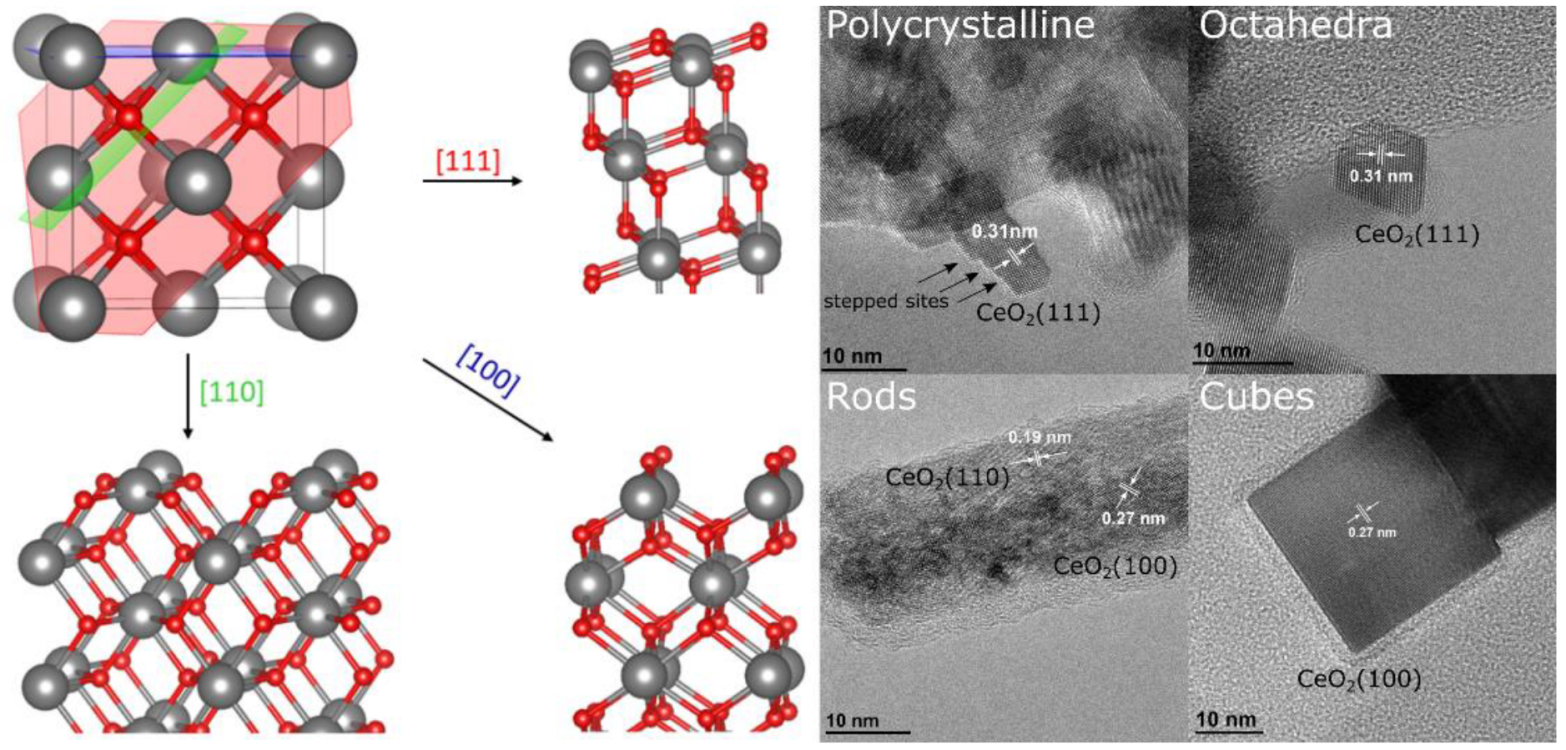
(Left) Overview of the different ceria facets. On the top left, the conventional unit cell of CeO_2_ is shown (Ce^4+^, gray; O^2–^, red). Furthermore, the [111] plane (red), the [100] plane (blue), and the [110] plane (green) can be seen. A cut through the ceria lattice along the direction perpendicular to those planes yields differently oriented ceria facets: CeO_2_(110) (bottom left), CeO_2_(111) (top right), and CeO_2_(100) (bottom right). (Right) Detailed TEM images of polycrystalline ceria (sheets), octahedra, rods, and cubes. The white arrows indicate the distance between the lattice planes in the direction of the particle surface.

The low-index (111), (110), and (100) surfaces of fluorite-type ceria (*Fm*3*m*) are shown in Figure 1. In the (111) facet, each atomic plane is charged but the repeat unit corresponds to an O–Ce–O trilayer, whereas in the (110) facet, each atomic layer contains a stoichiometric proportion of cerium and oxygen atoms. A cut through the ceria lattice along the ⟨100⟩ direction results in a surface with an excess of anions or cations (i.e., a polar surface). Such polar surfaces are not stable, and the polarity can be compensated for by the formation of oxygen or cerium vacancies, leading to a reconstructed surfaces, or by the adsorption of background gases, mostly leading to hydroxylated surfaces.^30^ The nature of the stable (100) surface reconstruction is a matter of debate.^32−35^ One of such reconstructions presents half oxygen monolayer termination, matching a checkerboard-like pattern (Figure 1), hereinafter referred to as a CeO_2_(100) facet.

The relative stability of the low-index ceria surfaces decreases in the following order: (111) > (110) > (100).^30−32,36^ Importantly, the surface properties of ceria depend on the exposed facet which has a different surface oxygen defect formation energy, *E*_vac,__O_. For instance, if we compare the calculated values (PBE+U/4.5 eV) for vacancy structures for which the closest distance between surface oxygen defects is comparable, *E*_vac,__O_ follows the (111) > (100) > (110) trend (*E*_vac_,_O_ = 2.27 eV (2 × 2), 1.82 eV *p*(2 × 2), and 1.32 eV (2 × 2), respectively).^1,2^

The facet-dependent properties are of importance in catalysis and can be exploited by using nanoshaped ceria such as octahedra, rods, and cubes. Engineering the shape of ceria particles offers a powerful tool for developing materials with enhanced catalytic properties.^37,38^ For example, polycrystalline ceria can be prepared by the thermal decomposition of cerium nitrate,^39^ which mainly terminates with the (111) surface due to its thermal stability. Octahedra, rods, and cubes can be synthesized by hydrothermal synthesis,^40,41^ and since the growth is kinetically controlled, less stable surfaces such as the (100) and (110) surfaces can also be obtained. The octahedra expose the (111) surface, the cubes the (100), and the rods the (110) as well as the (100). TEM images of such particles are shown in Figure 1, where in each case the distance of the lattice planes is indicated by white arrows. While the polycrystalline sheets and polyhedra terminate with the (111) surface, the sheets expose additional stepped sites.

## 3 Adsorption and Activation of Oxygen

**Figure 2:**
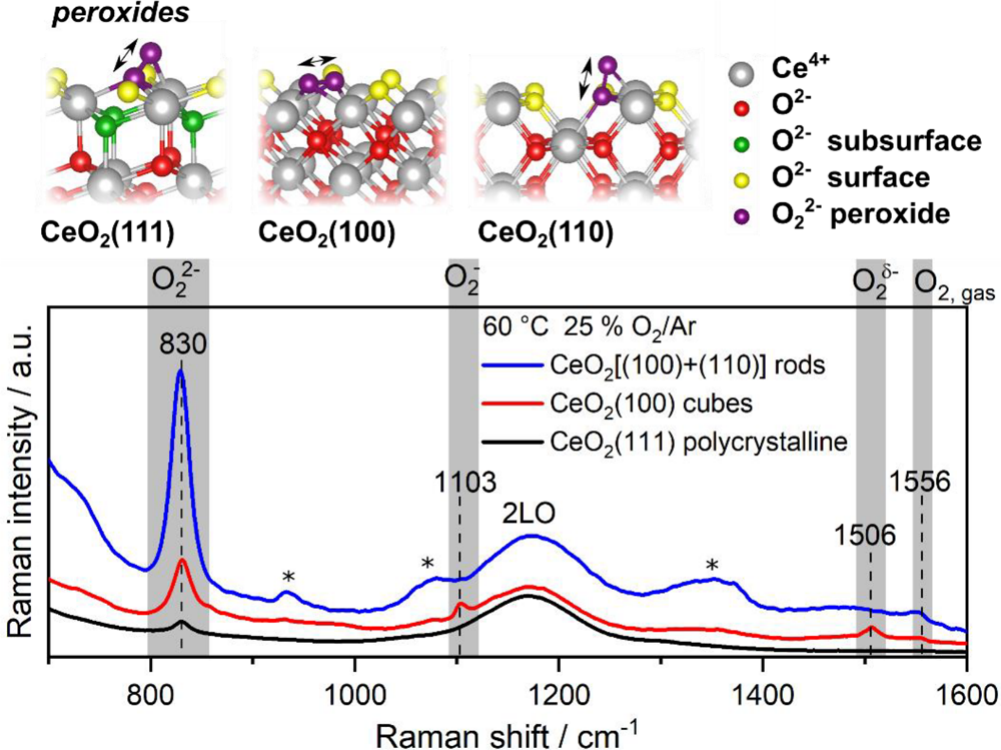
Atomic structure of the most stable peroxide species on (111), (100), and (110) ceria surfaces as calculated by DFT. The lower panel depicts the region of adsorbed oxygen species of *in situ* 532 nm Raman spectra of polycrystalline CeO_2_(111), CeO_2_(100) cubes, and CeO_2_[(110) + (100)] rods recorded in 25% O_2_/Ar at 60 °C with a total flow rate of 100 mL/min. Residues from the synthesis are marked (*).

The ability of ceria to reduce and oxidize (oxygen storage capacity) and its oxygen mobility play crucial roles in its catalytic applications, and thus an in-depth understanding of the O_2_ adsorption, activation, and dynamics is key to achieving improved catalytic efficiency. In this context, the exposed ceria surface facet has been shown to have a strong effect on the oxygen storage capacity of nanoshaped ceria.^37,42,43^ The reason is that oxygen activation at vacant surface oxygen sites, in the form of weakly adsorbed oxygen (O_2_^δ−^), superoxide (O_2_^–^), and peroxide (O_2_^2–^) species, is highly dependent on the ease with which surface oxygen defects can be created, which depends on the ceria facet exposed.^1^ Furthermore, after the initial oxygen activation, a complete dissociation of the adsorbed species with O atoms filling oxygen vacant sites can occur (i.e., oxygen is incorporated into the ceria lattice). For a detailed structural investigation of oxygen adsorbates on ceria surfaces, a combination of Raman spectroscopy and density functional theory (DFT) calculations has proven to be a powerful tool.^1−3,7,44^ As shown in Figure 2, peroxides (830 cm^–1^) are present on all facets, while weakly adsorbed oxygen (1506 cm^–1^) and superoxides (1103 cm^–1^) can be detected only on the (100) facet of the nanocubes. The absence of these two bands for the rods, exposing (100) and (110) facets, suggests that the coexistence of the two facets modifies the (100) facets, resulting in different properties as compared to those of ceria cubes.^2^

A closer inspection of the peroxide Raman bands in Figure 2 reveals a facet-dependent intensity as well as an asymmetry toward higher wavenumbers. The band is most intense for the rods and weakest for polycrystalline ceria. The observed behavior can be explained on the basis of different effects: the magnitude of the defect formation energy, the magnitude of the adsorption energy of O_2_^2–^ species, *E*_ads,O_2_^2–^_, which follows the trend (100) > (111) > (110) (*E*_ads,O_2_^2–^_ = −2.141 p(2 × 2), −1.919 (2 × 2), and −1.170 eV (2 × 2), respectively),^1,2^ the vibrational frequencies on the corresponding structures [(110) (898 cm^–1^) > (100) (868 cm^–1^) > (111) (855 cm^–1^)],^1,2^ and the surface area of the nanoshapes.

The low intensity on the sheets originates from the high defect formation energy on the (111) surface (as discussed above). Despite the high reducibility of the (110) facets, DFT shows that peroxides on (110) are less stable, likely decompose into the lattice,^2^ and possess the highest vibrational frequency, which we do *not* observe in the experiment. Consequently, the presence of peroxides on the rods can be attributed to the (100) facets. The higher peroxide intensity on the rods as compared to that on the cubes can then be explained by the large surface area of the rods, which is 3.5 times greater than that of the cubes^3^ while the fraction of (110) facets of the rods represents slightly more than half of their surface,^45^ and the larger number of intrinsic defects (caused by synthesis) in the rods; peroxide species will strongly adsorb to those surface defects. Thus, despite various contributions to the intensity behavior, a detailed understanding of adsorbed species becomes accessible by combined efforts from experiment and theory.

Furthermore, an examination of the stable peroxide species on the individual facets reveals that on the (111) and (110) facets, O_2_^2–^ species are oriented perpendicular to the surface, whereas on the (100) facet, O_2_^2–^species lie flat (Figure 2). Moreover, the O–O bond length is facet-dependent (1.445 Å (2 × 2)-(110) ≈ 1.446 Å (2 × 2)-(111) < 1.468 Å *p*(2 × 2)-(100)).^1,2^ The above-mentioned asymmetry of the peroxide bands originates from higher coverages of peroxides on the surface since as the peroxide coverage increases, the O–O bond length decreases, resulting in a blue shift of the vibration, which is observed for all low-index surfaces.^1,2^

Finally, it is of interest to consider the facet dependence of the oxygen storage mechanism. In this regard, the rods have been shown to possess superior properties^40,42,43^ compared to the cubes and polycrystalline ceria based on the better ability of the rods to incorporate oxygen into the crystal lattice upon dissociation of adsorbed peroxide species, which originates from the smaller distance between surface oxygen vacant sites on the (110) facet and the higher exothermicity of the reaction leading to the reoxidation of such a facet.^2^

## 4 Au/Ceria Catalysts: Support Participation and Structural Dynamics

Catalyst support materials are essential to stabilizing metal nanoparticles in many industrial processes. Common attributes of support materials are a high surface area, chemical stability, and the ability to disperse metal particles over the surface. Moreover, support materials may strongly influence the catalytic performance via metal–support interactions or even participation in the catalysis and may be divided into inactive (e.g., SiO_2_, Al_2_O_3_) and active (e.g., TiO_2_, CeO_2_) ones. Active support materials such as ceria are characterized by their reducibility and their direct participation in the redox cycle.

Ceria and ceria-based catalysts are known for their dynamic behavior upon variations in the gas environment, and thus the use of suitable *in situ*/*operando* approaches is required to capture the structural changes. Despite progress in the field, a detailed understanding of the structural properties of bulk and surfaces of ceria powders during reaction has been achieved only recently by combining Raman and IR spectroscopy with DFT, including vibrational frequency and intensity calculations. In the following text, the potential of such a combined approach for providing essential mechanistic information will be illustrated by the CO oxidation and WGS reactions over Au/ceria catalysts.

### 4.1 CO Oxidation

The low-temperature CO oxidation is of practical relevance but also an important prototype reaction in heterogeneous catalysis. The mechanistic details of the CO oxidation over ceria and in particular Au/ceria catalysts have been vigorously debated in the literature, including the role of the support and the nature of the active site.^38,47−52^

Starting with bare ceria, our recent study on differently shaped ceria nanoparticles has demonstrated their activity in CO oxidation at higher temperatures as well as the influence of the surface termination.^3^ In particular, the comparison of ceria rods, exhibiting CeO_2_(110) and CeO_2_(100) terminations, with ceria cubes with only the CeO_2_(100) termination reveals the superiority of the (110) facets over the (100) facets for CO oxidation, which is easily explained by the more facile formation of surface oxygen defects on the CeO_2_(110) facet (see above). Using the bare samples at a higher temperature (121 °C), CO conversions similar to those with gold-loaded samples (0.2 wt % Au) at a lower temperature (45 °C) could be obtained, highlighting that the reactivity of surface oxygen is a key aspect of the CO oxidation mechanism and that the presence of gold facilitates the availability of reactive surface oxygen by lowering the barrier for defect formation. In addition, CO adsorption is preferred on Au/ceria over the bare support, allowing gold to play a coordinating role in the course of the reaction. As will be discussed in the following text, a combination of recent *operando* spectroscopic and theoretical results has enabled us to develop a detailed mechanistic picture of room-temperature CO oxidation over Au/CeO_2_ catalysts.

**Figure 3:**
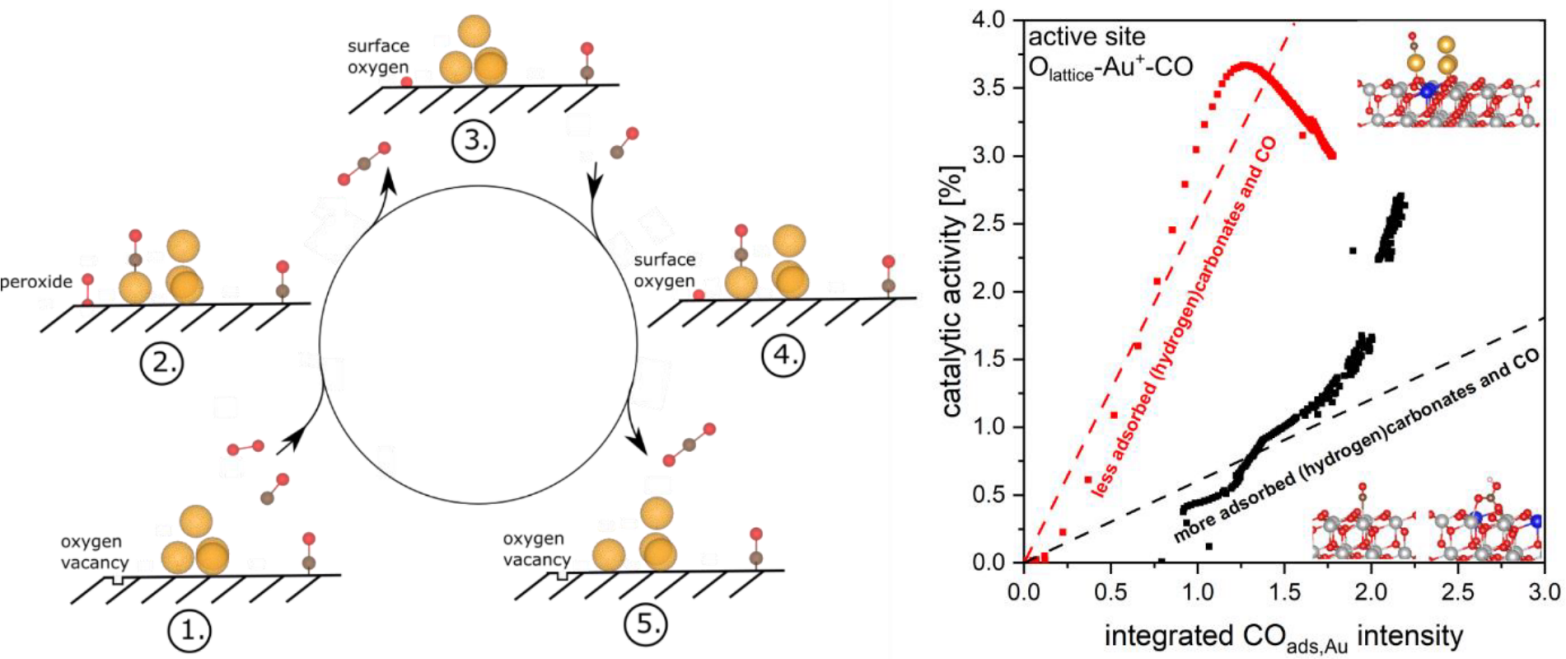
(Left) Proposed mechanism for the CO oxidation over Au/CeO_2_ catalysts based on combined *operando* spectroscopy and theory. (Right) The intensity of CO adsorbed on polycrystalline Au/CeO_2_(111) (2125–2130 cm^–1^) is initially correlated with the conversion of the Au/CeO_2_ catalyst for two pretreatments (equilibration in 25% O_2_ for 1 h at 21 °C, red; 1 h at 200 °C in 25% O_2_, black). The dashed lines are a rough interpolation indicating an initial relation between the adsorbed species and the conversion. The black line refers to a high and the red line refers to a lower concentration of adsorbed CO and (hydrogen)carbonate species. Data are from ref (13).

Regarding the mechanism of CO oxidation over Au/ceria catalysts (polycrystalline ceria), previous studies have shown that the Au–oxide interfacial perimeter plays a major role and that the activation of molecular oxygen occurs at the surface of the support via initial peroxide formation at ceria defect sites (see above).^53,54^ The latter was directly evidenced in our previous studies on a 0.5 wt % Au/CeO_2_ catalyst during room-temperature CO oxidation by using time-dependent *operando* Raman spectroscopy.^55^ As illustrated in Figure 3, molecular oxygen adsorbs onto a ceria surface oxygen vacancy in the vicinity of gold, leading to peroxide formation. The outer oxygen atom reacts with CO adsorbed onto gold, while the second oxygen atom fills the vacancy. Next, this lattice oxygen is consumed by reaction with adsorbed CO.^13,49,50^ Finally, oxygen vacancies are replenished by molecular oxygen, completing the catalytic cycle.

Very recent combined *operando* IR and theoretical results have elucidated further details about the catalyst dynamics and the state of the active gold, highlighting the role of cationic sites.^13,50,52^ For a detailed assignment of the experimentally observed IR frequencies, the CO adsorption on model Au/ceria catalysts, consisting of single Au_1_ and Au_4_ gold clusters adsorbed on a CeO_2_(111) surface, was studied by employing DFT calculations. Interestingly, the CO stretch frequencies (2125–2130 cm^–1^) under *operando* conditions are consistent with CO adsorbed onto both single isolated Au^+^ sites and/or pseudosingle sites in direct contact with the CeO_2_(111) surface. The latter refer to the gold ions that were slightly abstracted from a gold cluster, forming O_lattice_–Au^+^–CO species under the reaction conditions. After CO_2_ formation, it is energetically favorable for the abstracted gold ion to reintegrate into the gold cluster until further CO adsorption occurs. It is noteworthy that, independent of the pretreatment history (as-prepared or dehydrated, cf. Figure 3), the Au/ceria catalyst approaches the same state but after different times, underlining the structural dynamics of the catalyst in the presence of the reaction mixture. This observation can be attributed to the fact that at room temperature the formation of (hydrogen)carbonates (or adsorbed CO) on the bare support plays a role in blocking active sites, which is particularly noticeable in the dehydrated samples (black symbols in Figure 3). However, as the reaction continues, these spectator species can be displaced and a state of equilibrium is reached.^13^

The above combined *operando* and theoretical studies could resolve apparent differences in the literature regarding the nature of the active gold sites on low-loaded ceria, as metallic gold, interfacial gold atoms, isolated cationic gold ions, and mixtures of different sites had previously been considered.^56^ An interesting aspect concerns the role of water, which has been shown to facilitate CO oxidation by catalyzing the reaction of CO with OH groups, leading first to carboxyl formation followed by decarboxylation, as proposed both experimentally and theoretically.^57−60^ However, in such a scenario, the exact role of hydroxyl is still an open question, as *operando* IR spectra do not show a direct relation of the OH intensity changes to the activity.

#### 4.1.1 Facet Dependence

Because ceria directly participates in the catalytic reaction, it is of great interest to further explore the influence of the support on the mode of operation. In addition to above-mentioned facet-dependent differences in the ease with which oxygen vacancies can be created, the exposed facet may affect the interactions between ceria and the supported gold^23,61,62^ as well as between gas-phase molecules (CO, H_2_O) and the catalyst surface.^1,2,30,63^ Recently, we have examined structure–activity relations for CO oxidation over Au/rods (CeO_2_(110), CeO_2_(100)) and Au/cubes (CeO_2_(100)) using combined *operando* Raman/UV–vis spectra and DFT calculations,^3^ with the former showing higher low-temperature CO oxidation activity. In the following text, we will first illustrate important aspects of the facet-dependent behavior and then include our previous work on polycrystalline ceria supported gold in the discussion.^13,55,64^

**Figure 4:**
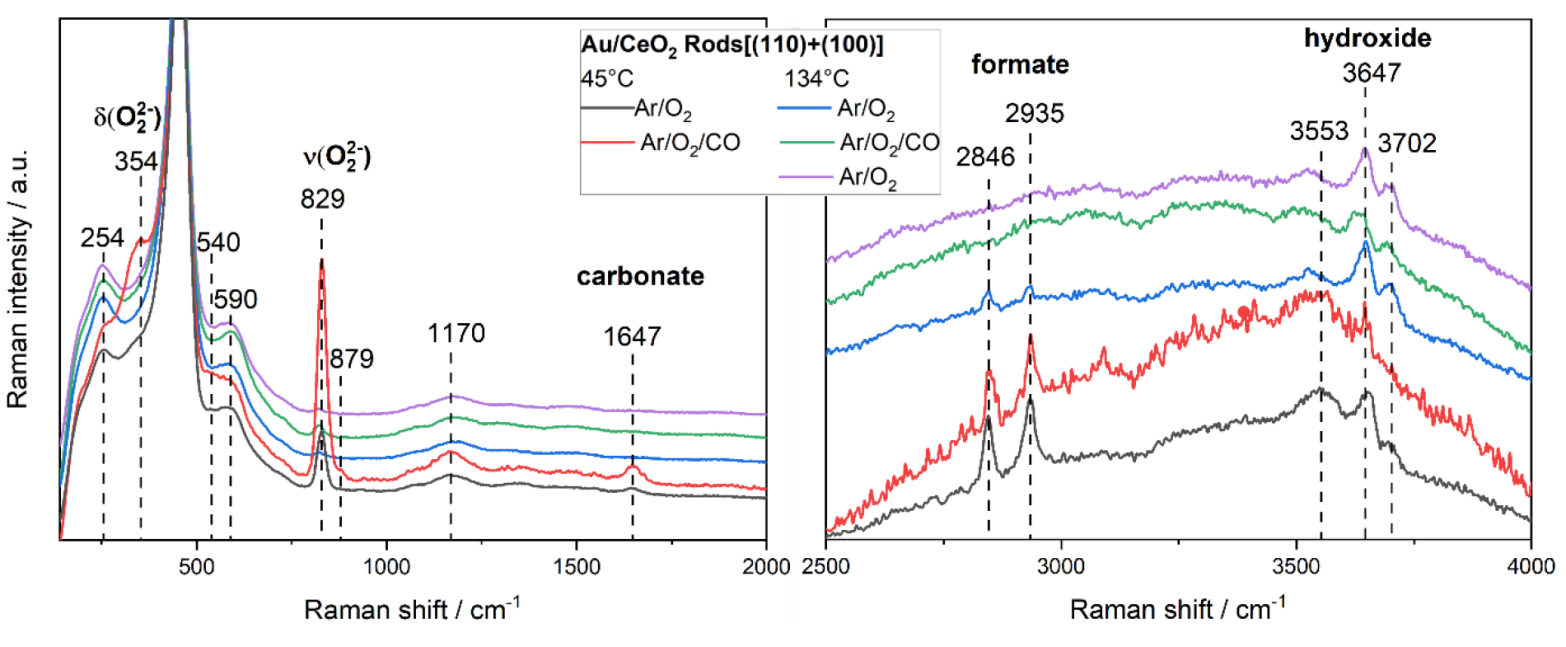
*In situ* and *operando* 532 nm Raman spectra of the low-wavenumber (left) and high-wavenumber (right) regions of gold-loaded ceria rods. Spectra were recorded at 45 °C/134 °C (flow rate: 100 mL/min) and feed compositions of 2% CO/25% O_2_/Ar and 25% O_2_/Ar for reactive and oxidative conditions, respectively. Spectra were offset for clarity. Data were taken from ref (3).

Figure 4 shows *in situ*/*operando* Raman spectra of Au/rods at low and elevated temperatures, containing characteristic solid-state phonons (254, 540, 590, and 1170 cm^–1^) and adsorbate-related features (354, 829, 1647, 2846, 2935, 3553, 3647, and 3702 cm^–1^). While details of the assignments based on DFT calculations of differently oriented ceria facets have been discussed elsewhere,^2,3,44^ we note that in the case of the Au/rods, switching from oxidative to reactive conditions at 45 °C results in a strong increase in the signals at 354 and 829 cm^–1^, i.e., those arising from peroxide species at surface oxygen defect sites (for high peroxide coverages).^2,3^ Furthermore, a subsurface/bulk reduction of the ceria support is observed by *operando* UV–vis spectra via an increased absorption at 570 nm (due to Ce^4+^–Ce^3+^ charge transfer).^3^ The larger changes in the intensity of the peroxide Raman bands as well as in the UV–vis absorption at around 570 nm for the Au/rods result from the easier reducibility of the CeO_2_(110) facets in the rods and can readily explain the higher catalytic activity of the rods in low-temperature CO oxidation.

At elevated temperatures, *operando* Raman spectra of Au/rods show a strongly decreased peroxide signal (Figure 4, left), indicating more facile peroxide dissociation and reaction with CO (see above). Regarding other adsorbate-related features, the carbonate band at 1647 cm^–1^ exhibits interesting behavior, increasing in intensity under the reaction conditions at low temperature but disappearing at higher temperatures (Figure 4, left).^3,55^ At low temperatures, we propose CO to react with lattice oxygen to form stable carbonate species, fully consistent with the theoretical results for CeO_2_(110) and CeO_2_(100) surfaces, showing carbonate formation to be highly exothermic.^4^ As a result, carbonate formation strongly inhibits CO oxidation at low temperatures because active sites are blocked. On the other hand, at elevated temperatures, the absence of the carbonate band indicates thermally induced carbonate decomposition (Figure 4, left).

Similarly, *operando* Raman spectra of the high-frequency region show formate-related bands at 2846 and 2935 cm^–1^, which decrease in intensity at elevated temperature and are no longer observed under the reaction conditions (Figure 4, right). By comparison with the corresponding spectra of the bare rods,^3^ we can conclude that gold promotes formate decomposition via proton transfer from formate species to surface oxygen, leading to hydroxide formation as evidenced in the *operando* spectra (Figure 4, right) as well as CO_2_ formation, as has been discussed previously for Pt/CeO_2_ systems.^60^

In contrast to the (100) and (110) facets in cubes and rods, the CeO_2_(111) surface does not support stable carbonate formation for geometric reasons which may favor CO oxidation reaction. On the other hand, the defect formation energy is largest for the (111) facet (see above). Interestingly, our polycrystalline ceria-supported gold catalysts have shown superior CO oxidation activity as compared to rods. Thus, besides facet-dependent effects, also the presence of stepped sites (Figure 1) needs to be taken into account to fully explain the reactivity behavior.^65^

### 4.2 WGS Reaction

#### 4.2.1 Facet Dependence

As an alternative to the industrial low-temperature WGS reaction catalyst Cu/ZnO/Al_2_O_3_, low-loaded noble metal-based catalysts (Pt, Au) on reducible oxides such as ceria have been suggested and shown to be highly active. However, important aspects such as the metal–support interaction and the state of the noble metal as well as the role of the ceria surface termination have not been unraveled. There is agreement on the participation of both the metal particles and the support material, but the detailed functioning of the catalyst has been subject to debate, as two types of mechanisms have been proposed in the literature (i.e., a redox mechanism and an associative mechanism). Recently, we have explored the influence of the surface termination on the performance and mode of operation of ceria-supported Au catalysts in more detail using combined *operando* spectroscopies and DFT calculations.^4,12^

**Figure 5:**
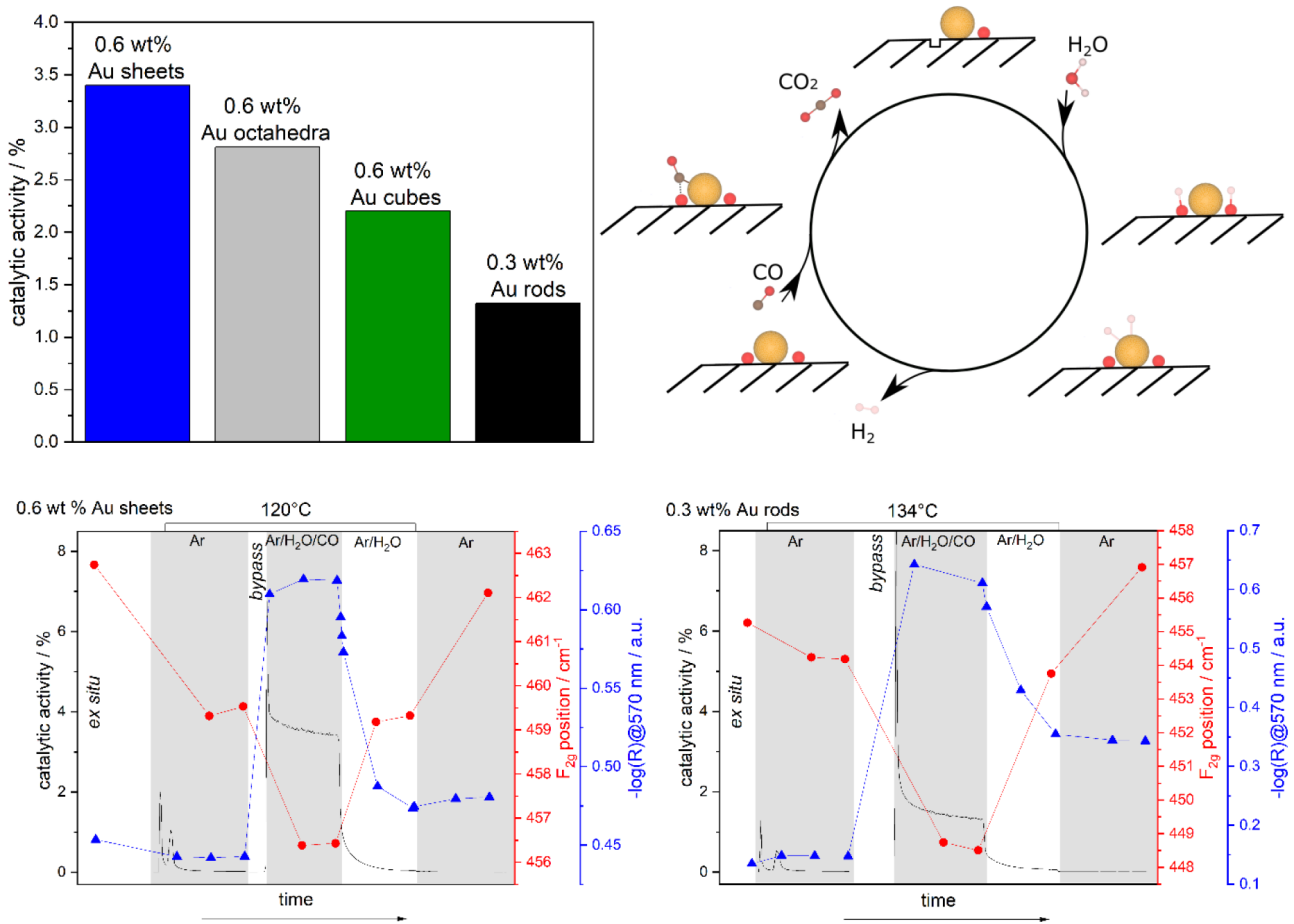
(Top left) CO conversion (in %) during the LT-WGS reaction of Au/ceria catalysts with different morphologies. The catalytic activity was measured after at least 1 h on stream at about 130 °C in 2% CO/8% H_2_O/Ar (flow rate: 100 mL/min). (Top right) Proposed mechanism for the WGS reaction over Au/CeO_2_ catalysts. (Bottom) *Operando* 532 nm Raman (red) and UV–vis (blue) results shown together with the catalytic activity (black) of 0.6 wt % Au/CeO_2_ sheets (left) and 0.3 wt % Au/CeO_2_ rods (right) during the WGS reaction (2% CO/8% H_2_O/Ar) at a flow rate of 100 mL/min. Prior to reaction, the catalyst was exposed to Ar, and after reaction, the catalyst was exposed to 8% H_2_O/Ar, followed by cooling to 48 °C in Ar. Data are from ref (4).

Figure 5 compares the catalytic activity of a series of Au/ceria catalysts with low gold loading using ceria sheets (polycrystalline, mainly CeO_2_(111)), octahedra (CeO_2_(111)), cubes (CeO_2_(100)), and rods (CeO_2_(110), CeO_2_(100)) as support materials. Please note that a higher Au loading further decreases the performance of the rods. A clear dependence on the ceria surface termination is observed, with polycrystalline ceria showing the best performance. To gain insight into the facet-dependent behavior, various aspects need to be considered, such as the stability of the ceria surfaces, their interaction with the reactants and products, and the Au–ceria interaction. Considering the latter, electron microscopy analysis has revealed the presence of highly dispersed gold, whereas a combination of UV–vis and XP spectroscopy indicated the presence of both metallic and cationic gold, with the fraction of metallic gold ranging from 70% for Au/sheets to 40% for Au/cubes.

Combined *operando* Raman and UV–vis spectroscopy allowed the exploration of the catalyst dynamics under the reaction conditions (Figure 5, bottom). In fact, *operando* UV–vis spectra show facet-dependent changes in the absorption at around 570 nm, which are related to Au plasmons and Ce^4+^–Ce^3+^ transitions.^66,67^ As shown exemplarily for polycrystalline ceria and ceria rods at the bottom of Figure 5, upon switching from reaction conditions (CO/H_2_O/Ar) to water (H_2_O/Ar) and finally an inert environment (Ar), the absorption significantly drops but does not return to the level observed prior to the reaction conditions. This behavior can be attributed to an agglomeration of gold particles during the WGS reaction, leading to an enrichment of neutral gold and thus an increase in plasmon absorption. These findings are consistent with observations made for gold particles supported on ceria–zirconia.^68^ Interestingly, the amount of absorbance increase for the two argon phases (i.e., before and after the reaction conditions) is smallest for sheets and largest for rods, thus showing an inverse trend compared to activity, strongly suggesting an influence of agglomeration on catalytic performance. Therefore, it is of great interest to maintain the metal dispersion on the support during the reaction. In this context, stable single-atom catalysts have recently been prepared by noble metal deposition onto CeO_2_–TiO_2_ or activated γ-alumina.^69,70^

To explore the support-related dynamics, we employed *operando* Raman spectroscopy. The bottom of Figure 5 shows the gas-phase-dependent position of the F_2g_ mode at around 450 cm^–1^, which at constant temperature is a quantitative measure of the changes in ceria stoichiometry. All Au/ceria catalysts show mode softening upon exposure to the reaction conditions, originating from a unit cell expansion due to the larger ionic radius of Ce^3+^ (Ce^3+^, 1.143 Å; Ce^4+^, 0.970 Å) formed upon ceria reduction (as supported by DFT).^44^ These observations are fully consistent with the *operando* UV–vis results (Figure 5), revealing a maximum 570 nm absorption under the reaction conditions, which besides gold surface plasmons (see above) is related to a reduction of the support due to charge-transfer Ce^4+^–Ce^3+^ transitions. On the other hand, neither the absolute F_2g_ shift nor the absolute absorption changes of the facet-dependent catalysts are directly related to the catalytic activity and to their facet-dependent defect formation energies, pointing to the fact that other aspects need to be considered besides reducibility, such as catalyst interactions with reactants (CO, H_2_O) and products (CO_2_, H_2_). Regarding the facet-dependent role of adsorbates, combined *operando* Raman and DFT studies provided new insights into ceria adsorbates, in particular, the role of carbonates. In fact, the less-stable surfaces (i.e., CeO_2_(110) and CeO_2_(100)) were shown to form stable carbonate species, which may block active sites and may therefore reduce the catalytic activity.

The proposed mechanism for LT-WGS over Au/ceria catalysts is summarized in Figure 5. As discussed above, CO oxidation takes place at the Au/ceria interface by the reaction between lattice oxygen and CO adsorbed on gold. As a result of the consumption of ceria lattice oxygen, an oxygen vacancy is created, which is proposed to strongly facilitate the dissociation of water. This latter step has been considered to be crucial for the feasibility of a redox mechanism.^12^ To this end, additional H_2_^18^O isotope exchange experiments provided evidence for the facile dissociation of water on Au/ceria catalysts.^4,12^ As the final step, H atoms from hydroxyl groups, located close to gold particles, recombine to molecular hydrogen over gold. In summary, the observed facet-dependent catalytic activity is attributed to a combination of active site blocking and Au agglomeration effects. This knowledge will facilitate the design of more active LT-WGS catalysts by focusing on the role of the support and engineering its properties toward enhanced gold stabilization.

## 5 Concluding Remarks

As illustrated above, the close interaction of *in situ*/*operando* Raman spectroscopy and theory represents a powerful approach to an atomic-level understanding of ceria and ceria-based catalysts. The influence of the surface crystallographic orientation on reactivity behavior has become accessible by the hydrothermal synthesis of ceria nanoparticles, which can be employed as working catalysts, thus bridging the material gap between idealized (single-crystal) and real catalytic systems. When this approach is applied, the ceria facet-dependent behavior is found to be specific to the reaction and can also be related to the facet-dependent reducibility, adsorbate stabilities, and gold–support interactions.

Generally, there have been an increasing number of combined Raman/IR and theory studies related to heterogeneous catalysts in recent years. As illustrated for the CO oxidation and the WGS reactions, combining *operando* vibrational spectroscopy with DFT calculations allows us to gain detailed insight into the mode of operation of ceria-based gold catalysts, including the participation of the support (sub)surface, oxygen dynamics, and specification of active sites.

Due to the overall complexity of heterogeneous catalysts, the application of other techniques (UV–vis, XAS, XRD, XPS, etc.) and their coupling with vibrational spectroscopy will be of great importance. Recent advances in vibrational spectroscopic techniques include shell-isolated nanoparticle-enhanced Raman spectroscopy (SHINERS) and tip-enhanced Raman spectroscopy (TERS), exploiting surface-enhanced Raman effects as well as transient techniques.^7^ On the other hand, the theoretical description of reduced ceria^31^ and the accurate determination of vibrational frequencies through the use of computational chemistry remain challenging for contemporary DFT methods,^71^ with the hybrid DFT methodology generally performing better.^72^ Combining highly sensitive vibrational spectroscopic techniques with highly accurate calculations will bring us closer to the ultimate goal of an atomic-level understanding of working catalysts, both spatially and temporally resolved.
